# CytoSorb removes MDMA in vitro, but is it an effective therapy in vivo?

**DOI:** 10.1186/s40635-020-00333-z

**Published:** 2020-07-29

**Authors:** Patrick M. Honore, Sebastien Redant, Thomas Datzmann

**Affiliations:** 1 ICU Department, Brugmann University Hospital, Centre Hospitalier Universitaire Brugmann, Place Van Gehuchtenplein, 4, 1020 Brussels, Belgium; 2 Universitätsklinik Ulm, Ulm, Germany

**Keywords:** 3,4-Methylenedioxymethamphetamine, Intoxication, Hemoperfusion, CytoSorb

## Abstract

**Background:**

3,4-Methylenedioxymethamphetamine intoxication can result in potentially lethal multi-organ failure, for which the current treatment is largely supportive. Recently, a report of the use of the CytoSorb device as a part of the successful treatment of a patient with 3,4-methylenedioxymethamphetamine intoxication and multi-organ failure has been described.

**Main body:**

While 3,4-methylenedioxymethamphetamine was very effectively removed by CytoSorb in vitro, the degree of removal in the clinical setting described may have been minimal. Indeed, the therapy was started relatively late in this case, and, as the therapy is concentration dependent, the removal of 3,4-methylenedioxymethamphetamine is likely to have been limited. On the other hand, in this case, CytoSorb hemoadsorption was very effective to treat rhabdomyolysis and hyperinflammation.

**Conclusion:**

The in vitro experimentation demonstrates that 3,4-methylenedioxymethamphetamine is effectively removed by CytoSorb. However, it is debatable whether the case report confirms the possibility of in vivo removal of 3,4-methylenedioxymethamphetamine by CytoSorb. Nevertheless, the potential of the CytoSorb device to contribute to the treatment of many critically ill patients has yet to be fully explored, and further studies are warranted.

## Introduction

3,4-Methylenedioxymethamphetamine (MDMA), known by the street names “Ecstasy” or “Molly”, is a popular illicit drug with stimulant and hallucinogenic properties. MDMA stimulates the release of serotonin, dopamine, and norepinephrine, which are responsible for both the desired and adverse effects of its ingestion. MDMA intoxication can result in potentially lethal multi-organ failure, for which the current treatment is largely supportive. In a recent issue of Intensive Care Medicine Experimental, Lang et al. reported the use of the CytoSorb device as a part of the successful treatment of a patient with MDMA intoxication and multi-organ failure [1].

## Elimination of MDMA in vivo

Typical recreational doses of MDMA have been shown to result in blood concentrations between 0.1 and 0.25 mg/L, with peak plasma levels occurring 2 h after ingestion [2]. Elimination of the drug from the body is moderately slow, with a half-life of approximately 8 h [2]. While the majority of cases of serious toxicity or fatality have occurred with blood levels between 0.5 and 10 mg/L, some have had levels as low as 0.11–0.55 mg/L [2]. The pharmacokinetics of MDMA have been shown to be non-linear, and toxicity may occur with small dose increases or with repeated doses due to saturation of enzymes responsible for its degradation [2, 3]. The actual composition of MDMA tablets varies greatly [2], and this is another factor that contributes to potential toxicity. Due to a low volume of distribution of MDMA and a moderate protein binding, the free proportion in the blood might be a valuable target for blood purification techniques.

## The CytoSorb device

Removal of drugs in the case of overdose or toxicity is a major challenge. Treatments such as intermittent hemodialysis and continuous renal replacement therapy (CRRT) can only potentially remove hydrophilic substances and as such are ineffective in the removal of lipophilic drugs such as MDMA [4]. Sorbent devices have been shown to effectively remove a wide range of drugs [4] and hold promise as a possible treatment in cases of intoxication.

The CytoSorb device is made up of biocompatible, highly porous polymer beads with a total surface area of approximately 40,000m^2^, resulting in a large capacity for clearance of substances from the blood via adsorption [5]. The hemoadsorption corresponds to the saturable fixation of molecules directly on a sorbent or along an affinity gradient depending on hydrophobic, ionic, and van der Waals interactions. An important characteristic of CytoSorb is that elimination is via passive diffusion, meaning that it will be efficient when the concentration of the substance to be removed is high, as early as possible after presentation.

## Elimination of MDMA in vitro

The in vitro capacity of the CytoSorb device to eliminate MDMA was investigated by Lang et al. by dissolving MDMA in fetal calf serum which was circulated in a custom-made system including a CytoSorb adsorber device [1]. After 5 min, the MDMA concentration measured distal of the adsorber device was non-detectable, indicating full removal of MDMA by the adsorber [1]. This experiment demonstrates that MDMA can be removed by CytoSorb, but this result is not directly comparable to the clinical setting, where many other blood components may compete with MDMA to be removed.

## Case report

In the case reported by Lang et al., the initial MDMA concentration, measured 16 h after admission, was 540 ng/mL (0.54 mg/L), a value within the toxic range [1]. CRRT was initially started to treat the acute kidney injury with a moderate mean dialysis dose of 30 mL/kg/h. Thereafter, the CRRT circuit was expanded to include Cytosorb hemoadsorption due to rhabdomyolysis and hyperinflammation for 7 days (using 7 devices in total). Taking into account the half-life of 8 h, and the reduced liver metabolism in this patient, we can postulate that the initial level (16 h earlier, i.e., two half-life periods of 8 h) may have been as high as 1500 ng/mL. Serial measurements revealed a decline in serum MDMA concentrations from 540 to 140 ng/mL within the first 24 h after adsorption therapy was started [1]. This is in keeping with the expected decline in the MDMA level due to metabolism, which, given the half-life of 8 h, would result in a level of 130 ng/mL at 24 h.

## Conclusions

The in vitro experimentation demonstrates that MDMA is effectively removed by CytoSorb. However, it is debatable whether the case report confirms the possibility of in vivo removal of MDMA by CytoSorb. The documented decline in MDMA levels could be simply explained by the expected pharmacokinetics of the drug in a healthy individual, though it has to be acknowledged that the peak MDMA level, extrapolated from the first measurement at 16 h following admission, indicates that the patient had a significant intoxication, and the consequent multi-organ failure may have resulted in a lower elimination capacity. The initiation of CytoSorb therapy was most likely beneficial for cytokine removal, myoglobin removal, and liver support, but its contribution to MDMA removal may have been minimal.

Nevertheless, the potential of the CytoSorb device to contribute to the treatment of many critically ill patients has yet to be fully explored and further studies are warranted.

## Data Availability

Not applicable.

## Abbreviations

MDMA: 3,4-Methylenedioxymethamphetamine
CRRT: Continuous renal replacement therapy
